# EpiMINE, a computational program for mining epigenomic data

**DOI:** 10.1186/s13072-016-0095-z

**Published:** 2016-09-29

**Authors:** SriGanesh Jammula, Diego Pasini

**Affiliations:** Department of Experimental Oncology, European Institute of Oncology, Via Adamello 16, 20139 Milan, Italy

**Keywords:** ChIP-seq, RNA-seq, Chromatin immunoprecipitation, NGS, Quantification, Correlation

## Abstract

**Background:**

In epigenetic research, both the increasing ease of high-throughput sequencing and a greater interest in genome-wide studies have resulted in an exponential flooding of epigenetic-related data in public domain. This creates an opportunity for exploring data outside the limits of any specific query-centred study. Such data have to undergo standard primary analyses that are accessible with multiple well-stabilized programs. Further downstream analyses, such as genome-wide comparative, correlative and quantitative analyses, are critical in deciphering key biological features. However, these analyses are only accessible for computational researchers and completely lack platforms capable of handling, analysing and linking multiple interdisciplinary datasets with efficient analytical methods.

**Results:**

Here, we present EpiMINE, a program for mining epigenomic data. It is a user-friendly, stand-alone computational program designed to support multiple datasets, for performing genome-wide correlative and quantitative analysis of ChIP-seq and RNA-seq data. Using data available from the ENCODE project, we illustrated several features of EpiMINE through different biological scenarios to show how easy some known observations can be verified. These results highlight how these approaches can be helpful in identifying novel biological features.

**Conclusions:**

EpiMINE performs different kinds of genome-wide quantitative and correlative analyses, using ChIP-seq- and RNA-seq-related datasets. Its framework enables it to be used by both experimental and computational researchers. EpiMINE can be downloaded from https://sourceforge.net/projects/epimine/.

**Electronic supplementary material:**

The online version of this article (doi:10.1186/s13072-016-0095-z) contains supplementary material, which is available to authorized users.

## Background

 All cells maintain their identity through a robust genomic organization. Different activities that modify the chromatin environment, more commonly defined as epigenetic, play critical roles in preserving and using the genetic information. This involves a series of modifications that influence the genome at different levels, starting from changes at single nucleotides (i.e. DNA methylation and its oxidized forms) to the regulation of higher orders of DNA organization. To unravel the molecular mechanisms behind each layer of regulation, previously developed techniques, such as chromatin immunoprecipitation (ChIP), expression of different forms of RNAs and bisulphite conversion, have been coupled to high-throughput sequencing (seq) technologies. Of all, ChIP-seq is most widely used for mechanistic studies, where immunoprecipitations are coupled with chromatin formaldehyde cross-linking and high-throughput sequencing to map the location of any specific epitope along the genome in living cells. Similarly, RNA-seq is another standard experimental approach for profiling genome-wide gene expression in any sample of interest. Thus, the conjunction of ChIP-seq and RNA-seq analyses allows the direct impact to be studied for given factors or specific modifications in regulating transcription. These milestone technical improvements have led to an exponential increase in the availability of data in public repositories. However, functional analyses performed with these data are often restricted to experimental observations.

Before making any biological inferences from the data generated through high-throughput sequencing platforms, sequencing data have to undergo a series of computational analyses, which can be regarded as primary and secondary analysis. Primary analysis mainly involves aligning sequencing data to a reference genome. Numerous programs are designed for this and are very well optimized. For instance, programs like BWA [1] and Bowtie [2] are commonly used for ChIP-seq data alignment, while others like TopHat [3], PALMapper [4] and STAR [5] are used for RNA-seq data alignment. To complete primary analyses, ChIP-seq aligned data are further processed to determine regions of enrichment along the genome, thus identifying potential DNA binding of a target protein or the deposition of histone post-translational modifications (PTMs). This process is known as peak calling, and MACS [6] and SICER [7] are some commonly used applications. At the same time, RNA-seq aligned data are processed to measure the levels of expression of different genes. Level of expression is studied in terms of reads per Kb per million (RPKM) or fragments per Kb per million (FPKM), programs like ERANGE [8], TopHat [3] and RSAT [9] can be used for this. In the recent years, this part of analysis was well established and often supported by sequencing facilities. This makes processed data commonly available and not a bottleneck for users. Upon publication, these data become further available in these formats in public domains like GEO [10], thus becoming available to the entire scientific community.

Depending on the biological questions to be addressed, further downstream analysis can be very different from one another. In particular, ChIP-seq data can be further processed in many different ways, such as mapping ChIP-enriched regions to the closest gene, and complete gene sets can be annotated to determine which biological process/molecular functions or biochemical pathways are enriched. GREAT [11] application is specifically designed for annotating such regions of interest. Another common task is to identify highly represented motifs in enriched regions, applications like MEME-ChIP [12] or Pscan [13] are more commonly used for this purpose. Similarly, RNA-seq data from different experiments can be further processed specifically to identify differentially expressed genes. Cuffdiff2 [14], DESeq2 [15] and edgeR [16] are the most commonly used tools for identifying differentially regulated genes.

In terms of usage, different programs are designed to make it easy for all users to carry out computational analysis. The only systems that render specific programs in a linked pipeline are Galaxy [17] and Cistrome [18]. However, the level of complexity related to epigenomic dynamics makes such limited analysis insufficient to address complex biological questions involving a large number of multiple datasets. The vast amount of data available from different studies presents an opportunity for exploring these relationships at a much deeper level, with the potential of better characterizing genome-wide dynamics and exposing hidden layers of regulation. To achieve this possibility, programs with in-build analytical and data mining methods, power for supporting bulk-processed data from different disciplines, are needed.

Although primary and extended analyses (i.e. ChIP-seq peak calling) are mature and broadly available, programs with above-mentioned capabilities are still not available, which thus restricts the analytical power to highly experienced computational biologists. With such approach tools like SeqMINER [19], and some utilities of HOMER [20], Cistrome [18] provides provisions for quantitative and correlative analysis, which remain restricted to a limited framework. However, these tools are very limited, as they cannot handle multiple samples, link changes within with transcription, predict dependencies, filter datasets on the basis of their relevance, identify features to characterize samples or perform differential analysis. Moreover, programs like HOMER [20] and Cistrome [18] cannot deal with raw aligned data but rather require processed aligned data, adding a further layer of complexity.

In addition to these stand-alone applications and command line tools, several packages have been designed in R for similar purpose, such as RepiTools [21] for analysis of enrichment-based epigenomic data, ChIPpeakAnno [22] for annotating enriched regions, DiffBind [23] for identifying differentially regulated regions between experiments, and many others available in bioconductor. The main disadvantage of using R packages and other command line tools is that it requires prior knowledge of programming. From an experimental biologist point of view, this could mean that even small tasks are tedious.

We were interested in developing a light-weight stand-alone open source application with genome-wide analytical features. We designed EpiMINE, a computational program for mining epigenomic data. The application is designed to be easy to use, with minimal input files, so that any experimental biologist with minimal computational background could use it easily, thus reducing dependency on others. This application is available with graphical user interface (GUI) and command line facility. The GUI option would make the application extremely user-friendly for scientists with no computational background. EpiMINE excludes primary/secondary analysis due to the extensive availability of resources for these analyses. Rather, it uses data from primary/secondary analysis as input for further downstream analysis, based on standard data that are easily accessible from public domain. EpiMINE has several utilities, each with its own analytical capabilities for genome-wide correlative and quantification studies. Depending on the biological query, different utilities can be executed sequentially.

## Results

We built EpiMINE with different utilities, each with distinct functionalities. In brief, it harbours the following utilities: *ENRICH*, which allows it to determine preferential enrichments at multiple sets of regions of interest (ROI) among annotated datasets (bed files); *CoREG*—performs co-localization analysis; *MCOR*—defines correlations between multiple datasets (bam files); *TCOR*—determines correlations between two datasets (bam files); *QIRI*—quantifies different datasets (bam files) within specific ROI; *QARI*—quantifies different datasets (bam files) around ROI; *PMS*—generates profiles using different datasets (bam files) across ROI; *TDIFF*—performs differential analysis among two groups; *MDIFF*—performs differential analysis among two or more groups; *ABRI*—predicts dependencies between datasets in the form of bed files; *VarSEL*—identifies meaningful datasets out of many (bam files) to describe specific ROI; *CLASS*—performs classification analysis; *MatHM*—generates heatmaps from the output of the other utilities; *extBAM*—extends reads by any desired length in pre-existing bam files.

To highlight the different features of EpiMINE, we took advantage of processed ChIP-seq and RNA-seq data generated by the ENCODE consortium [24] from different human cell lines to postulate biological scenarios and analyse the obtained results.

### Enrichment

**Fig. 1:**
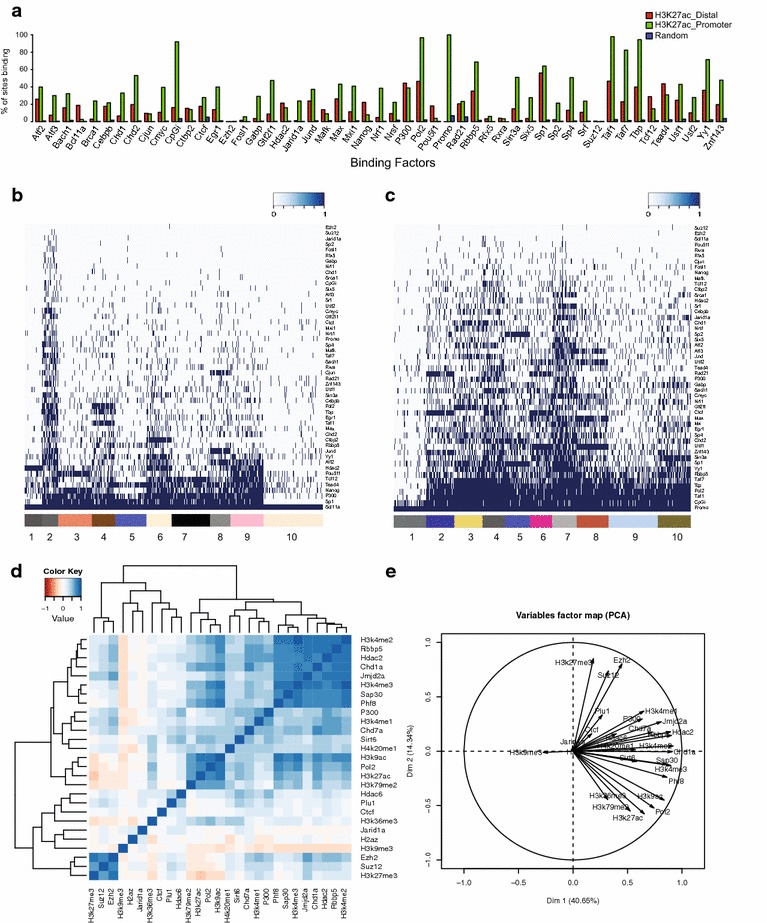
Preferential enrichment, coexistence and correlation analysis. **a** Barplot representing proportion of H3K27ac-positive promoters (in *green*), H3K27ac-positive enhancers (in *red*) and random regions (in *blue*), bound by different factors. **b** Heatmap showing the presence (*dark blue*) or absence (*light blue*) of different factors in a Bcl11a-binding regions. Closer the presence of any factor to Bcl11a, greater the coexistence. **c** Heatmap showing the presence (*dark blue*) or absence (*light blue*) of different factors in promoters of the top 3000 highly expressed genes. Closer the presence of any factor to Promo, greater the coexistence. **d** Genome-wide correlation between different factors along all promoters of the human genome. **e** Variable plot with different factors and their degree of correlation with others along all promoters of human genome across first two principal components

For most epigenomic analysis, determining whether the ROI show any preferential enrichment towards any known set of annotated regions is essential. In such situations, the *ENRICH* section of the program is useful. For instance, we were interested in determining whether a set of different factors, for which we have obtained ChIP-seq location data, can preferentially bind active promoter or enhancer elements in human embryonic cells (H1hESC). The genomic location of active promoters or enhancers can be easily determined by the accumulation of H3K27 acetylation (H3K27ac) with respect to a mapped transcription start site (TSS). Using ENRICH, we took into consideration H3K27ac-enriched regions in H1hESC and separated these regions into two broad categories: (1) regions residing in close proximity to promoters (±2.5 kb from TSS) and (2) regions lying away from promoters. This analysis identified bona fide active promoters (*n* = 4600) and enhancers (*n* = 2033) in H1hESC. These two sets of regions were used to determine the levels of association of 49 different factors for which ChIP-seq results were generated by the ENCODE consortium in H1hESC cells. This analysis showed that peculiar sets of factors clearly help in defining enhancers from promoters (Fig. 1a).

The results of this analysis are shown in Fig. 1a, which represents the proportion of ROI being bound by each individual factor. Apart from the analysis with input files, this utility provides the option to include random regions in the analysis (Fig. 1a, blue bars). This allows the extent of significance of this comparative analysis to be determined with respect to its random occurrence. This analysis clearly showed that active regions are all completely devoid of repressive factors, such as Ezh2 and Suz12. Importantly, components of the transcription machinery, such as RNA-POLII, TBP and TAF1, were present in both active promoters and enhancers, while factors such as Gabp, Brca1, Nrf1, Six5, Sp2, c-myc and Gtf2f1 were specifically enriched at active promoters. In contrast, DNA-binding TFs, like Oct4 (Pou5f1) and Nanog, were preferentially enriched at active enhancers, as previously reported [25, 26]. Interestingly, this unbiased analysis allowed us to identify Bcl11a and Tcf12 as novel factors specifically associated with active enhancer regions.

Using the *CoREG* utility, we further investigated whether factors that are specifically enriched at enhancers coexist together or not. This utility helps to dissect the extent of co-regulation between different factors based on the absence or presence of a given factor in each ROI. Using all Bcl11a-enriched regions as a reference, we found that Bcl11a frequently co-localized with the enhancer-specific TFs Nanog, Pou5f1, Tead4 and Tcf12, as well as with more promiscuous factors such as P300 and Sp1 (Fig. 1b). When the same analysis was performed using a set of promoters corresponding to the top 3000 highest expressed genes in H1hESC, this set of factors was indeed not enriched (Fig. 1c). Hence, this analysis strongly suggested that the novel enhancer-associated factors Bcl11a and Tcf12 co-regulate *cis*-regulatory regions together with Nanog, Tead4 and Pou5f1 in ES cells, highlighting the power of these new analytical tools.

### Quantification and correlation

Most genome-wide location studies generate multiple large ChIP-seq datasets, for which a major task is determining the extent of correlation among multiple datasets to identify closely related datasets that, by clustering together, highlight convergent or divergent biological behaviours. This type of analysis is facilitated with the *MCOR* section of the program, which can take multiple datasets and perform correlations at a genome-wide level or along specific ROIs. To illustrate this tool, we scanned the behaviour of 27 different factors from H1hESCs with respect to all human promoters. We subjected the datasets to two distinct correlation methods: Pearson’s correlation (Fig. 1d) and principal component analysis (PCA; Fig. 1e). In both types of analyses, the results identified two types of clusters: a repressive cluster marked by a strong correlation between Polycomb proteins (Suz12 and Ezh2) and their related histone PTMs (H3K27me3), and factors and histone PTMs associated with active transcription (H3K27ac, H3K9ac, Pol2, H3K79me2). With respect to the Pearson correlation, PCA provided much more extended information. First, the angle of separation allows a lack of any relationship between datasets representing active versus repressive features to be depicted. Second, the profile of H3K9me3 deposition strongly diverged from all other datasets consistent with its well-established deposition in constitutive heterochromatin. Third, the arrow length for each dataset provides information related to the contribution of each factor. For instance, the limited lengths of H2AZ, Ctcf and Jarid1a highlight their minimal contribution to defining promoter elements.

### Comparative quantification and its effects

A great challenge of ChIP-seq analysis is to move from qualitative information about the location of a given factor or modification along the genome towards more quantitative information between multiple experimental conditions in relation to other biological outcomes, such as changes in transcription. This implies more complex computations that also take into consideration intrinsic biases related to the sequencing procedure. To capture these changes, we designed quantitative methods that can identify such changes among multiple datasets and relate them with expression information (when provided). To exemplify our tool, we portrayed different scenarios to show how different ways of quantification can be experimentally meaningful.

**Fig. 2:**
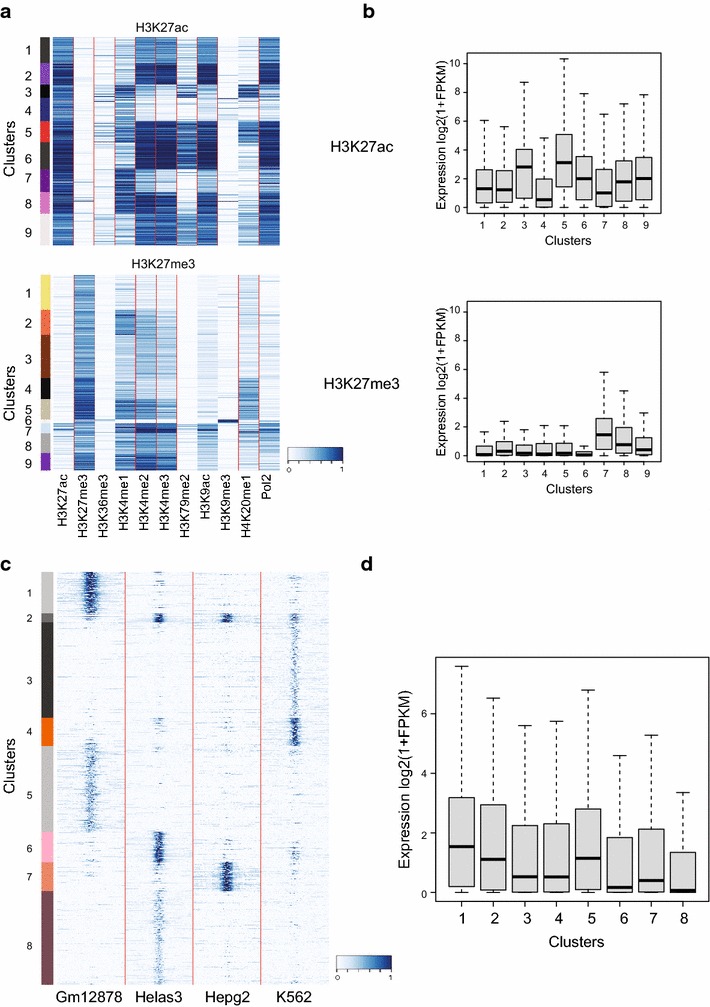
Quantification in and around ROI. **a** Heatmap with genome-wide-based normalized intensities for different histone modifications RNA-PolII in H3K27ac (*top panel*)- and H3K27me3 (*bottom panel*)-positive regions. **b** Expression level of target genes in each cluster identified in **a**. *Top panel* represents expression levels for target gene clusters for H3K27ac regions; *lower panel* represents H3K27me3-positive regions. **c** Intensities of H3K27ac ChIP in a 5-kb region surrounding the centre of enhancer regions, across five different *cell lines*. **d** Expression levels of target genes in Gm12878 in clusters identified in C

As a first case study, we used two samples of H1hESC—one representing a set of H3K27ac-enriched regions (active transcription; *n* = 6633) and the other representing a set of H3K27me3-enriched regions (repressed transcription; *n* = 5406)—to determine the deposition behaviour of several other histone PTMs along these two functionally different sets of genomic regions. For this, we provided to *QIRI* program with ChIP-seq datasets comprising 10 different histone PTMs together with RNA polymerase II and complemented this with H1hESC gene expression data, for all genes with their respective FPKM values in log2 form. The program processes data, computes the quantification and presents results in a form that can be visualized as a heatmap, with H3K27ac-enriched regions shown in the upper panel and H3K27me3-enriched regions in the lower (Fig. 2a). Each row of the heatmap represents one ROI. To uncover specific patterns within each cluster, data can be subjected to either hierarchical or k-means clustering. For the present analysis, data from each sample were subjected to k-means with nine clusters. Clustered data were then used to explore for specific expression patterns. The program associates each ROI to the closest gene and represents the expression distribution of all genes associated within each cluster as boxplots (Fig. 2b), allowing immediate visual comparisons of the results. This analysis clearly showed that all H3K27ac target genes present a higher level of expression than H3K27me3 target genes, consistent with their respective roles in activating and repressing transcription.

Within H3K27ac-enriched regions, clusters 3/4/7 identified active enhancers marked by the presence of H3K27ac and H3K4me1 and by the absence of H3K4me3 deposition. Other clusters identified active promoters, which are marked by H3K27ac and high levels of H3K4me3. Interestingly, the closest genes to cluster 3 enhancers, which contain higher levels of H4K20me1, H3K79me2 and RNA polymerase II, displayed a higher level of expression with respect to clusters 4 and 7, which contained a lower level of deposition for these modifications.

The results related to H3K27me3-enriched regions identified clusters 5/7/8/9 as representing bivalent domains [27, 28], which are marked by the presence of both H3K27me3 and H3K4me3. Within these clusters, cluster 7/8 presented high levels of RNA-PolII association with respect to clusters 5/9 and is characterized by a higher level of expression of its associated genes. This result showed that deposition of repressive marks is not sufficient to exclude transcription and highlights the requirement of quantifying multiple types of datasets in parallel to stratify the functional status of transcriptional regulatory regions. In agreement with this, it is important to note that the small set of genes linked to cluster 6, which are marked by the co-deposition of both H3K27me3 and H3K9me3, has undetectable transcription levels, showing that acquisition of H3K9me3 locks H3K27me3-repressed genes in a transcriptionally non-permissive status.

These quantifications can be based on a genome-wide level or can be applied to specific ROIs. A genome-wide approach normalizes, quantifies and scales the ChIP-seq signals along the entire genome, while ROI-selection performs the same quantification but only taking genomic regions of ROIs into account. In the genome-wide approach, quantification is processed in small bins, then the bins representing each ROI are merged and the mean signal is reported. If the analysis is restricted to a set of ROI, quantification and scaling will be specifically applied to this frame. It is important to note that it is always advisable to perform genome-wide analysis to capture true intensities, since it is possible that by quantifying signals with respect to a restricted set of genomic regions, the intensities could result in over- or under-representation with respect to a quantification that takes into account the entire range of signals along the genome. To avoid such biases, our program supports genome-wide-based quantification.

Further quantification studies are important in determining the extent of spread of the signal with respect to each ROI. Signal spread can be investigated over complete ROI or only in surrounding regions by extending from centre of each ROI. *QARI* utility facilitates such analysis. To elucidate this option, we took into consideration active enhancers from four different tissues—lymphoblastoid (Gm12878), leukaemia (K562), liver carcinoma (Hepg2) and cervical carcinoma (HeLa-S3)—to determine the spreading of the H3K27ac signal over a 10-kb region. All enhancers from individual tissues were merged together and submitted to the program along with gene expression data specific to Gm12878. QARI utility extends to a selectable fixed length (default 5-kb up- and downstream) from the centre of each region and further segments these regions into small bins of 50 bp length (set as default). The H3K27ac computed from the four different tissues was quantified, scaled and subjected to K-means clustering with *k* = 8. Results can be visualized as heatmap (Fig. 2c). The analysis clearly segregated tissue-specific enhancers, giving cluster 1/5 highly specific to lymphoblastoid, clusters 3/4 specific to K562, clusters 6/8 specific to Hela-S3 and cluster 7 specific to Hepg2, plus a cluster (cluster 2) that seems to represent a small set of constitutive enhancers present in all four tissues (Fig. 2c). Interestingly, there are sets of enhancers within each tissue with higher levels of H3K27ac with respect to others. In Gm12878, the levels of H3K27ac in cluster 1 are much higher than in cluster 5. The same applies to K562 and Hela-S3. It is possible that the clusters with highest levels of H3K27ac may represent super enhancer regions as previously reported [26]. Consistent with this, when the results were related to the expression of the closest genes in Gm12878, the expression of genes associated with Gm12878-specific enhancers (clusters 1/5) was significantly higher than in other clusters, thus representing active enhancers in different tissues (Fig. 2d).

A further advantage of this tool is the possibility to combine data from different experimental conditions. This becomes particularly useful when the same factor or modification is used in different experimental conditions (i.e. using the same antibody). In such a case, the program applies a global scaling over all datasets; otherwise, scaling is applied only within each individual dataset. The pros and cons of these approaches can be appreciated in Additional file 1: Figure S2A and S2B. Additional file 1: Figure S2A is the same as Fig. 2c, where the program by defaults assumes that all datasets are handled independently, irrespective of which antibody was used; in contrast, in Additional file 1: Figure S2B, this option was turned on, and all datasets are scaled together.

**Fig. 3:**
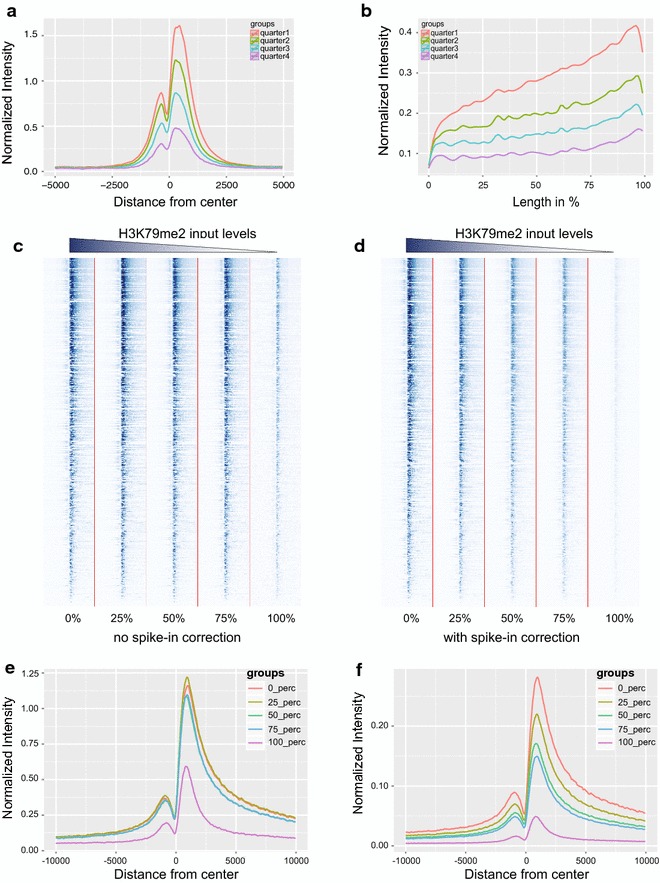
Average profile and spike-in-based normalization. **a** Average profile of H3K4me3 in promoter regions of genes classified on the basis of expression levels (*high to low*). **b** Average profile of H3K36me3 in gene bodies of genes classified on the basis of expression levels (*high to low*). **c** Intensities of H3K79me2 within 10 kb surrounding TSS (both up- and downstream) in regions possessing H3K79me2 in WT samples, and its fate in other samples induced with different levels of inhibitor without a reference genome. **d** Intensities of H3K79me2 around 10 kb surrounding TSS (both up- and downstream) in regions possessing H3K79me2 in WT samples, and its fate in other samples induced with different levels of inhibitor with a reference genome. **e** Average profile of H3K79me2 within 10 kb surrounding TSS (both up- and downstream) in regions possessing H3K79me2 in WT samples, and its fate in other samples induced with different levels of inhibitor without a reference genome. **f** Average profile of H3K79me2 within 10 kb surrounding TSS (both up- and downstream) in regions possessing H3K79me2 in WT samples, and its fate in other samples induced with different levels of inhibitor with a reference genome

Previous tools have allowed the levels of different ChIP analyses to be quantified over individual regions. However, we were also interested in determining the general genome-wide behaviour of a specific factor/modification along different set of regions or experimental conditions. In such cases, composite profiles of different ChIP analyses become simple and highly informative. In our program, we supported such analysis with the provision of quantifying target(s) over complete ROI (with either invariable length or over a constant region from the centre). *PMS* utility is specifically designed for this. Taking advantage of expression data from H1hESC cells, we sorted the genes on the basis of their expression levels (high to low) and partitioned gene expression into quarters. The first quarter represents highly expressed genes, while lower quarter represents low or not expressed genes. We then quantified the H3K4me3 and H3K36me3 levels at both promoters and gene bodies for the genes belonging to each individual quarter, respectively (Fig. 3a, b). For H3K4me3, we quantified its levels with respect to promoter centres (i.e. centred on the TSS with the surrounding region extended by a constant length of 5 kb up- and downstream of the TSS). Each region was further broken in smaller bins of 50 bp to quantify the signal over length. Similarly, we quantified H3K36me3 levels within the gene bodies of each individual gene. In this case, due to the invariable gene length, each gene was subdivided into defined finite blocks, whereby each block represents a fixed proportion of the total length of each gene body. These data were averaged within each quarter and plotted together (Fig. 3a, b). It is well established that gene expression, the levels of H3K4me3 deposition at promoters and the accumulation of H3K36me3 within gene bodies are positively correlated [29, 30]. Indeed, our analysis perfectly validated such behaviour (Fig. 3a, b). Genes belonging to quarter 1 displayed higher levels of both H3K4me3 at promoters and H3K36me3 within gene bodies with respect to genes with a lower expression level (quarter 2/3/4; Fig. 3a, b). One of the advantages of this analysis is that the program makes use of strand information (when provided), which helps to make sense of the data. For instance, it can be easily observed from our results that H3K4me3 deposition preferentially occurs towards the +1 nucleosome, aiding proper positioning and active transcription. In same way, H3K36me3 levels are higher towards the gene terminal portion. The differences of not using strand information can be appreciated in Additional file 1: Figure S2C and S2D.

This tool also supports quantification based on “spike-in” data. Recent reports have shown that data generated through standard ChIP-seq procedures do not capture real changes in histone PTM deposition, particularly when the overall global levels of a specific modification vary between experimental conditions due to technical biases of the ChIP-seq procedure [31]. To circumvent this technical problem, a standard ChIP-seq can be combined with a spike of the same chromatin from other reference genomes. This new procedure is able to better quantify the levels of a target PTM among different experimental conditions at each specific ROI. To prove the power of this option, we analysed data generated for H3K79me2, using different amounts of chromatin in which H3K79me2 is either present or absent. These chromatin spikes were added in different proportions (0, 25, 50, 75, 100 %) to mimic a linear reduction in global H3K79me2 levels. Data were analysed by considering or not the presence of an equal amount of exogenous Drosophila reference chromatin [31]. This analysis clearly shows the lack of linearity of standard ChIP-seq analyses and highlights the quantification power of the spike-in correction (Fig. 3c–f).

### Differential quantification

**Fig. 4:**
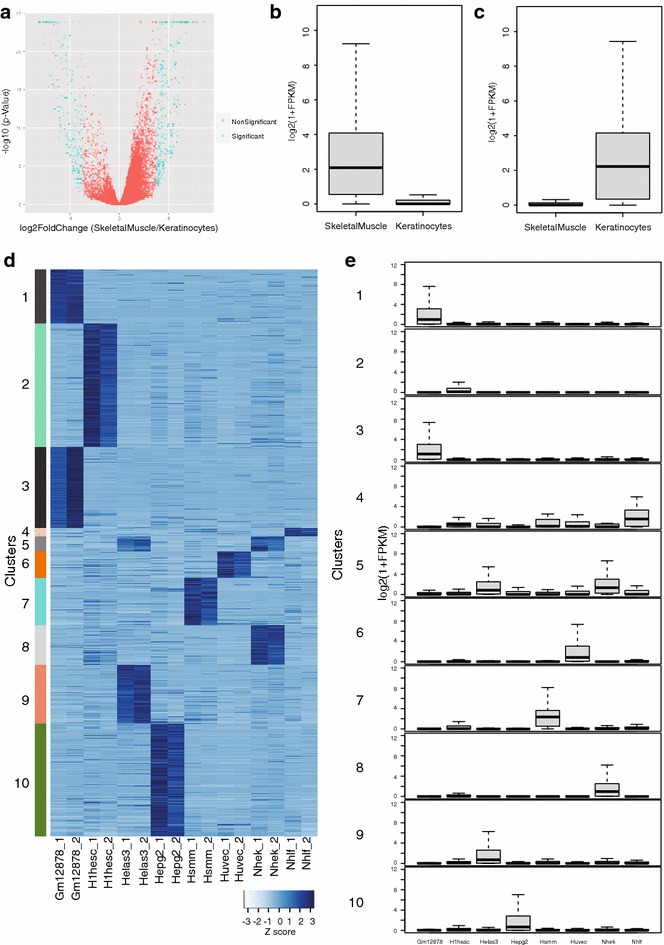
Differential analysis. **a** Volcano plot representing significantly enriched promoters (marked in *green*) harbouring different levels of H3K4me3 methylation in skeletal muscle as compared to keratinocytes. **b** Distribution of expression levels of genes with promoters that show significantly higher levels of H3K4me3 in skeletal muscle as compared to those of keratinocytes. **c** Distribution of expression levels of genes with promoters that show significantly higher levels of H3K4me3 in keratinocytes as compared to those of skeletal muscle. **d** Significantly enriched promoters with respect to K4me3 across eight different cell lines. Represented here are their intensities in standard z-score form. **e** Expression level of target genes in each cluster across eight different cell lines identified in **d**

Quantification-based differential studies can also help to identify markers, allowing to differentiate between two or more cell types. For instance, we questioned whether the deposition pattern of the same histone PTM in two different tissues could be used to distinguish one tissue from the other. To test this, we chose H3K4me3 ChIP-seq data (a marker of active transcription in gene promoters) from skeletal muscle (Hsmm) and keratinocytes (Nhek). We applied differential analysis over all promoters for H3K4me3 deposition. Using computed normalized read intensities, the *TDIFF* utility program identified a set of genes that were significantly enriched for H3K4me3 through a Fisher’s test in skeletal muscle (*n* = 512) over keratinocyte (*n* = 406; Fig. 4a). When we provided expression data for all genes in these two tissues, the program linked all enriched promoters to their respective target genes. This analysis clearly showed that the expression levels of H3K4me3 target genes in their respective tissue were significantly higher than others (Fig. 4b, c). This was further confirmed by performing tissue specificity with DAVID [32] using the output files containing the list of promoters significantly enriched in either skeletal muscle or keratinocyte. Both lists showed higher specificity towards their respective tissue, validating the tissue specificity of our results (Additional file 1: Figure S2E, S2F).

Similarly, situations arise in which differentially enriched regions need to be detected not only between two independent systems but also across multiple systems, a possibility that is included in our tool. To show its functionality, we extended the biological logic of Fig. 4a over multiple tissues, with the aim of identifying tissue-specific markers. Based on normalized read intensities, the *MDIFF* utility of the program identified differentially enriched H3K4me3 promoters across all datasets through a chosen statistical test (ANOVA or Kruskal–Wallis). All statistically significant regions were represented in the form of a heatmap, whereby the normalized read intensities were transformed to standard *z*-scores (Fig. 4d). To identify tissue-specific patterns, results were subjected to k-means clustering, using *k* = 10. This analysis clearly showed that all tissue-specific, differentially enriched H3K4me3 promoters were clustered together (Fig. 4d). To further cross-validate that these promoters are true markers of tissue specificity, the program also linked all promoters with the expression of their respective genes across all tissues within individual clusters. This resulted in the distribution of the expression of all target genes within individual clusters (Fig. 4d). Comparing the results side by side, we confirmed that genes associated with tissue-specific promoters indeed displayed tissue-specific expression (Fig. 4d, e). For example, cluster 1 represents promoters that are specific for lymphoblastoid cell line (Gm12878), which indeed display greater expression levels with respect to all other tissues. Similar conclusions can be applied for all other clusters.

### Probabilistic relationships

**Fig. 5:**
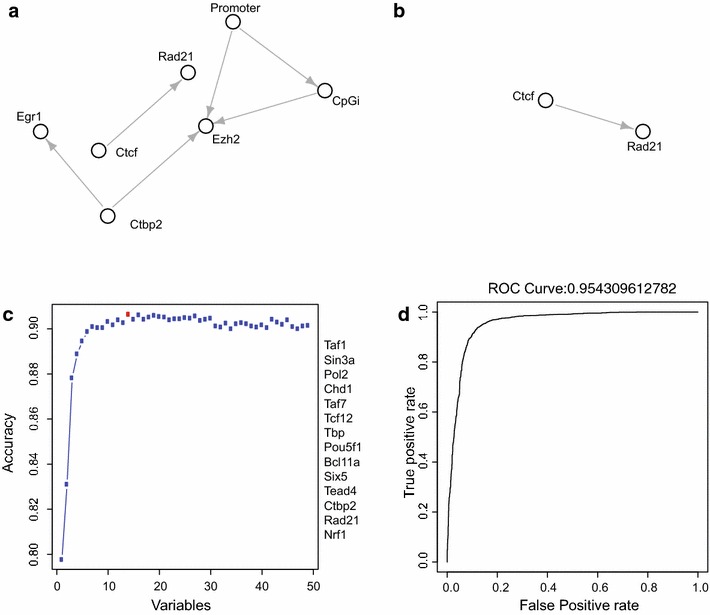
Predicting dependencies and characterizing ROI. **a** Bayesian network showing dependency between different factors in compact chromatin regions of genome presided by Suz12. **b** Bayesian network showing dependency between different factors in random regions of genome. **c** Plot signifying the accuracy of different set of variables for characterizing active enhancers and promoters. **d** ROC curve representing strength of an SVM-trained model for classifying active enhancers and promoters, using variables with a high accuracy level identified in **c**

Exploring relationships between different chromatin-associated factors, with the aim to better dissect the role of each entity and its functional contribution in a given biological process, requires increasing availability as well as the capability of generating large sets of genome-wide location analysis. We designed a utility that helps to predict the probabilistic relation between different factors, either at a genome-wide level or specifically within ROIs, taking advantage of a Bayesian network approach. To introduce this analysis, we wanted to determine which factors localize at genomic regions of compact chromatin and, among these, which factors showed dependency on each other. We selected regions enriched for Suz12 (*n* = 4789), a component of the Polycomb repressive complex 2 (PRC2) and an established marker of compact chromatin, to compute its localization with respect to defined genomic features and other DNA-binding factors that could represent a common functionality. For this, we took the binding sites of 51 different DNA-binding factors along with two sets of annotated genomic regions, of CPG island (CpGi) and gene promoters. These data are then processed by the *ABRI* utility by implementing a learning algorithm, in which either constraint or scoring analysis can be performed, depending on the users interest (see “Methods” for further details). In our analysis, we used a constraint-based grow shrink algorithm in an iterative bootstrap process, where 70 % of total data were selected randomly, and constructed a Bayesian network from this. This step was repeated 500 times, and only the dependency factors that were identified in 95 % of the networks were retained to generate a final dependency network (Fig. 5a). To further determine the validity of the generated network, we repeated the above process using random regions of the same input size (*n* = 4789) to generate a “control” network (Fig. 5b). Comparison of both networks clearly identified that the dependency between Ctcf and Rad21 was not specific for the Suz12 bound regions, while the rest of the dependencies were shown to be specific (Fig. 5a, b). Indeed, a functional relationship between Ezh2 and Suz12 (Fig. 5a) and its preferential localization at CpG-rich genomic regions at gene promoters (Fig. 5a) are well established. In addition, this analysis identified novel specific dependencies between Ezh2, Ctbp2 and Egr1 that were never reported previously. To test whether the dependency between Ezh2/Ctbp2 is valid, we used the ChIP-seq profiles of these proteins to determine their association among all Suz12 binding sites. Indeed, we found that Ctbp2 occupies nearly half of the Suz12-binding sites (Additional file 1: Figure S2G). This observation has been very recently validated experimentally, showing a functional link between the activities of Ctbp2 and PRC2 [33].

### Classification

Recent studies have shown that different loci of the genome display precise epigenetic characteristics. In terms of regulation, classifying genomic regions on the basis of their epigenetic characteristics is helpful for classifying distinct roles. Here, we present a system in which different classes can be segregated on the basis of quantifying different factors (which can be either TFs or histone PTMs). In this tool, we supported classification based on a support vector machine (SVM) approach, employing both linear and nonlinear models of classification. This system can be used for either training or with a combined training and prediction processes. In the training scheme, the program takes into consideration all provided datasets and lists the performance as an area under ROC with accuracy scores. If the user is satisfied with the classification on the training data, the classification model can be further applied to a new set of ROIs. In addition to the classification, the program also supports pre-data analysis. Such an approach is recommended in cases dealing with large numbers of datasets. The user has to judge which datasets contribute the most for the classification. In such situations, users can choose a pre-selection analysis, which subjects all datasets to a recursive feature elimination process in which all possible subsets are considered, and accuracies for all variable sizes are reported. The program also generates a list of predictors with the highest accuracies, which can be further used for the classification.

As an example, we show the process of characterizing active enhancers and active promoters marked by H3K27ac in ES cells on the basis of 49 different TFs and other regulators. Initially, all 49 datasets were subjected to the *VarSEL* utility for a variable pre-selection process. This process helps to eliminate the lower contributing datasets. From the results, we observed that a combination of 14 datasets was sufficient for classifying active enhancers and active promoters (Fig. 5c), while including additional datasets did not give major improvements. Therefore, through pre-selection analysis, we were able to downsize the number of datasets for further analysis, thus removing noise and reducing computing power. From available literature, we can easily assure that all critical factors known to characterize active promoters and enhancers were selected (Fig. 5c). Interestingly, the novel enhancer-specific factor that we identified in this analysis, Bcl11a, was among the new selected datasets, further validating its enhancer specificity (Fig. 1a). As a final validation, we achieved an ROC of 0.95 when all these 14 datasets were fed to *CLASS* utility for classification process (Fig. 5d).

All presented analyses demonstrate the versatility of EpiMINE in interpreting epigenomic studies with multiple datasets and experimental conditions integrating location with expression results. In addition to these main features, EpiMINE is packed with additional tools that are not presented in our figures due to space constrains but that are well described in the download package. These include: (1) a time-saving tool, *MatHM*, which generates heatmaps from already generated results in different forms without rerunning complete analysis; (2) *extBAM*, which extends aligned reads to a certain fixed length; (3) *TCOR*, which determines the correlation between two datasets.

EpiMINE is available in two different forms: one is designed to support an old version of bedtools (2.16/2.17) and the other is designed to support latest version of bedtools (2.25). EpiMINE with support of bedtools (version 2.16/2.17) can be downloaded directly from the following link: https://sourceforge.net/projects/epimine/files/EpiMINE_bedtools_2.16_2.17.tar.gz/download, while EpiMINE with support of bedtools (version 2.25) can be downloaded directly from following link: https://sourceforge.net/projects/epimine/files/EpiMINE_bedtools_2.25.tar.gz/download. We recommend the users to use the older version of bedtools (version 2.16/2.17) as it provides much better performance as compared to that with higher version. The installation process will automatically recognize the presence of a newer version, uninstall it and reinstall the appropriate bedtools version.

All the described analyses were performed on Mac OS with 4 GB of RAM. The performance of each individual utility is summarized in Table 1. All these analyses were performed with EpiMINE with the support of bedtools version 2.16. This table describes the time taken by each utility for completing each analysis together with the number of datasets/ROIs that were processed for each specific task, highlighting the high feasibility of performing these types of analyses also form a local laptop machine.

**Table 1:** Performance of different utilities of EpiMINE in generating results mentioned in this manuscript along with number of files processed

Mac Book laptop—Mac OS 10.11—4 GB RAM						
Figure	Utility	Real	User	Sys	Number of bam files processed	Number of bed files processed
1A	ENRICH	3m4.420s	1m40.539s	0m8.173s		51
1B, C	CoREG	2m16.439s	1m7.569s	0m3.969s		51
1D, E	MCOR	74m41.883s	69m40.924s	2m19.813s	27	1
2A, B	QIRI	26m27.703s	23m25.298s	0m47.114s	12	2
2C, D	QARI	240m28.371s	182m38.537s	39m15.460s	8	1
3A	PMS	287m16.025s	240m14.324s	22m8.330s	2	4
3B	PMS	125m56.986s	119m0.587s	4m43.368s	2	4
3F	PMS	62m1.064s	45m49.599s	9m50.006s	5	1
4A, B, C	TDIFF	4m14.759s	3m8.678s	0m3.862s	2	1
4D, E	MDIFF	37m15.644s	35m2.737s	0m50.188s	36	1
5A	ABRI	8m11.794s	6m5.741s	0m7.175s		51
5C	VarSEL	143m28.846s	143m28.846s	3m25.513s	49	2
5D	CLASS	29m0.011s	26m41.527s	0m44.029s	14	2

## Discussion

The fast development of NGS technologies has radically changed experimental approaches in “wet labs” leading to the generation of a surplus of high-quality data, which are also available from public resources. To handle this increasing amount of data, in terms of both size and complexity, we developed a platform, EpiMINE, with efficient analytical methods. We tested to which level of flexibility it can be applied for different epigenomic studies. This development is an attempt to open a new window for high-throughput data analysis, providing a platform with useful methods for genome-wide studies. The uniqueness of the program lies in handling and analysing the changes within and/or across multiple samples against different datasets and their flexible linkage of results to expression data. Each utility of the program generates all necessary results, different plots of good resolution and many other supplementary text files. Supplementary text files can be helpful for further downstream analyses, which can be used as input to other utilities of the EpiMINE program, thus increasing its flexibility without the need of additional modifications. Depending on the utility, the program offers some additional features for enhancing the results, including options for smoothing data, making use of strand information for analysis and selecting colour for heatmaps (a detailed description can be found in the help of utility and manual of EpiMINE). This program comes with both graphical and command line utilities, allowing it to be used by also by non-specialist users with very minimal computational background. It can be executed in both Mac OS and Linux operating systems.

Taking advantage of publicly available human ENCODE datasets and analysing them using EpiMINE, we have cross-verified some known observations to show the power and accuracy of EpiMINE. At the same time, we generated novel findings, such as the preferential association of the Bcl11a transcription factor at active enhancers with respect to promoters and of the association between Suz12 and Ctbp2 in chromatin compact regions of human embryonic stem cells.

We tried to avoid redundancy of running alignment, peaking calling and others, and we specifically chose to restrict the program on tertiary analysis. These steps are now very much standardized, and many pipelines have been well established for doing such tasks. In addition, many sequencing facilities provide support by default for both primary and secondary analysis. The most limiting step is to handle further downstream analyses based on experimental design. With few exceptions, no other program is as capable as EpiMINE in performing comprehensive genome-wide analyses with multiple datasets. Table 2 represents a comparison between the various features provided by EpiMINE with respect to other well-known tools, such as Cistrome [18], ChIPseeqer [34], HOMER [20], seqMINER [19], diffReps [35] and macs2 bdgdiff (https://github.com/taoliu/MACS).

**Table 2:** Comparison of different features of well-known programs with EpiMINE

User interface	seqMINER	HOMER	ChIPseeqer	Cistrome	EpiMINE
	GUI	CL	GUI	WEB GUI	GUI/CL
Standard analyses					
Peak calling	FALSE	TRUE	TRUE	TRUE	FALSE
Gene ontology	FALSE	FALSE	TRUE	FALSE	FALSE
Motif analysis	FALSE	TRUE	TRUE	TRUE	FALSE
Peaks based analysis					
Enrichment of different samples in ROI	FALSE	FALSE	FALSE	FALSE	TRUE
Coexistence of different samples in ROI	FALSE	FALSE	FALSE	FALSE	TRUE
Correlation based on peaks	FALSE	FALSE	TRUE	FALSE	FALSE
Predicting dependencies based on peaks	FALSE	FALSE	FALSE	FALSE	TRUE
Introducing and analysing random regions	FALSE	FALSE	FALSE	FALSE	TRUE
Quantification-based analysis					
Number of samples can be processed in a run	1	1	NA	≥1 (applicable for few cases)	≥1 (applicable for all cases)
Works with raw data (no processing required)	TRUE	FALSE	FALSE	FALSE	TRUE
Signal within ROI	FALSE	FALSE	FALSE	FALSE	TRUE
Spread of Signal around ROI	TRUE	TRUE	FALSE	TRUE	TRUE
Profile generator	TRUE	TRUE	FALSE	TRUE	TRUE
Genome-wide/ROI-specific correlation	FALSE	FALSE	FALSE	TRUE	TRUE
Introducing and analysing random regions	FALSE	FALSE	FALSE	FALSE	TRUE
Provision of k-means clustering on quantified data	TRUE	Not in build	FALSE	TRUE	TRUE
Provision of hierarchical clustering on quantified data	FALSE	Not in build	FALSE	FALSE	TRUE
Correlating clustered data with expression	FALSE	FALSE	FALSE	FALSE	TRUE
Spike-in normalization	FALSE	FALSE	FALSE	FALSE	TRUE
Predicting dependencies based on peaks	FALSE	FALSE	FALSE	FALSE	TRUE
Filtering datasets on the basis of their importance wrt ROI	FALSE	FALSE	FALSE	FALSE	TRUE
Classification studies	FALSE	FALSE	FALSE	FALSE	TRUE

User interface	macs2 bdgdiff	diffReps	EpiMINE
	CL	CL	GUI/CL
Differential studies			
Identifying differential regions between two conditions without replicates	FALSE	TRUE	TRUE
Identifying differential regions between two conditions with replicates	TRUE	TRUE	TRUE
Identifying differential regions between more than two conditions with replicates	FALSE	FALSE	TRUE

*GUI* graphical user interface, *CL* command line, *TRUE* feature is available in tool, *FALSE* feature is not available in tool, *NA* feature is not applicable for tool, *Not in build* feature should be executed outside the tool

## Conclusions

EpiMINE is a light, user-friendly application for epigenomic studies, which has the capability of handling multiple datasets and performing different types of analyses that are either ROI restricted or genome wide.

## Methods

EpiMINE can be downloaded from https://sourceforge.net/projects/epimine/. It constitutes several utilities, each with its own capabilities. They are dedicated for enrichment, coexistence, quantification, correlation, differential, predicting dependencies and classification studies. Here, we describe its design, file formats and the different methods it uses for analysis.

### Design and dependencies

The EpiMINE program is developed in the Python platform. It requires the presence of Python (version ≥2.7) with the following modules: wxpython, pysam, pybedtools, rpy2, R application with gplots, ggplot2, RColorBrewer, FactoMineR [36], kernlab [37], bnlearn [38], igraph, fastcluster [39], caret, ROCR [40] packages and bedtools [41] installed. wxpython is used for graphical user interface, pysam for processing alignment files and rpy2 for statistical analysis through R. This program is supported in Mac OS and Linux. It is available both in form of GUI and command line with support in both Mac OS and Linux. To facilitate end-user with installing any missing dependencies, program is bundled with automated script, which can check for any missing dependencies and install them accordingly.

### Datasets

All presented results were generated using human ENCODE [24] data. We used histone modification (HM), transcription factor and expression datasets of human embryonic stem cells (H1hESC), lymphoblastoid (Gm12878), umbilical vein endothelial cells (HUVEC), cervical carcinoma (HeLa-S3), liver carcinoma (HepG2), leukaemia (K562), skeletal muscle fibroblast (HSMM), human lung fibroblast (NHLF) and epidermal keratinocytes (NHEK). For effective comparison between different tissues expression, data were subjected to quantile normalization. For spike-in analysis, published data were downloaded [31] and aligned to a human or *Drosophila* reference genome, and bam files were generated for further use in downstream analyses.

### Input files

Each utility requires input data, which can be a bed, bam or genome file. (1) bed files are tab-separated files containing information certain locus of genome. More details about the format can be obtained from https://genome.ucsc.edu/FAQ/FAQformat.html#format1. (2) bam files are standard binary format files containing details about the alignment of sequencing data with reference genome. (3) Genome files are also tab-separated files, with two columns listing chromosomes and their lengths.

#### Samples

Multiple bed files containing regions of interest (ROI), where one bed implies one sample.

#### Datasets

Either bam/bed files, against which the single/multiple samples can be analysed.

#### Promoters

We considered promoters as regions 2.5-kb both up- and downstream from the TSS of a given gene.

#### Active promoters

We considered these as promoter regions that are positive for H3K27ac.

#### Active enhancers

We considered these as regions that are distant from promoters and have both H3K27ac and H3K4me1 but not HK4me3.

#### Use of bedtools

Bedtools is used for computing overlaps, coverage in regions of interest, and for finding closest transcript.

### Random regions

To improve analytical power, the program can generate random data (if enabled) in bed format and analyse these data in parallel to the main data stream. Random regions are generated of similar size as that of input data by shuffling the genomic coordinates and chromosomes of input regions. Reordered coordinates are then cross-checked with reference genome file in order to verify that the new coordinates are within the limits of chromosome length.

### Quantification

Different utilities of EpiMINE like TCOR, MCOR, QARI, VarSEL and CLASS perform computational analysis on tag intensities quantified from different datasets. In such cases, program preforms genome-wide quantification in which genome is fragmented into small bins of user defined length. Program then counts total number of reads within each bin which is then normalized to sequencing depth. In cases where an input/control sample is provided, normalized reads for input dataset are computed within each ROI and are then subtracted from normalized reads of target datasets. If spike-in data are provided, then normalization is carried out in a similar manner as explained previously [31]. To avoid any skewness in data distribution, normalized intensities are log transformed. To efficiently compare different datasets derived either with similar or different antibodies, quantification is subjected to scaling. If datasets are generated with the same antibody (an option provided in program), the whole quantification will be scaled to 0–1. On the other hand, if datasets are generated with different antibodies, the individual datasets are scaled to 0–1 separately to allow liable comparison between datasets. Scaling can be explained better by considering matrix (*X*) containing *n* rows and *m* columns, where each row represents one ROI and each column represents each dataset. If all *m* datasets are generated with same antibody, then scaling is performed such that minimum and maximum values of the matrix are set to 0 and 1. If on the other hand all *m* datasets are generated through different antibodies, then scaling is performed such that minimum and maximum values for each column of the matrix are set to 0 and 1. In cases where target ROI is provided, then the bins representing target ROI are retrieved. If single ROI represents multiple bins, then the average of all bins for that particular ROI is considered. Complete flow of quantification is schematically represented in Additional file 1: Figure S1.

Results from quantifications can be further used to identify patterns of quantification of different datasets over all ROI by subjecting it to hierarchical/k-means clustering. Further, clustered results can be linked with expression data. If expression data are provided, then each ROI is assigned to the closest gene, and the expression distribution of target genes across different clusters is presented as a boxplot.

This program harbours other utilities, such as QARI and PMS, that performs quantification around a ROI from its centre or can be profiled over a ROI. In such cases, the ROI or its extended form is fragmented into smaller bins based on the user’s preference, and normalized reads are computed for these bins. Quantification can be represented for each individual ROI or as an average profile over all ROIs.

### Correlation

Using genome-wide quantification (as explained above), a correlation between different datasets can be computed either by Pearson’s product–moment correlation, rank correlations (Spearman, Kendall) or principal component analysis (PCA) methods. Once any of the three initial methods is chosen, the correlation between all possible dataset pairs is computed and transformed into a correlation matrix. This matrix is then represented as a heatmap, in which the degree of correlation is associated with a colour code. If PCA is chosen, the program generates a variable graph with a circle of correlation across the first two principal components capturing maximum variance from the data. Variable graph signifies the degree of closeness/relatedness between multiple datasets, where each dataset is represented as an arrow. Variable graph can be interpreted at different levels. First, the amplitude of the angle between two arrows is directly linked to the degree of correlation, whereby the smaller the angle between two datasets, the higher their correlation. A 90° angle signifies no correlation, while an opposite angle (>90°) reflects an anti-correlation between two datasets. Second, the length of the arrow represents the importance of that dataset in representing whole data, whereby the longer the length, the greater the importance of that variable, and vice versa.

### Differentially enriched regions

For any given couple of datasets where each dataset represents one condition, differentially enriched ROIs are discovered using Fisher’s test. For individual ROI, *p* value is computed from 2*2 contingency table where columns represent datasets and rows represent normalized intensities for datasets inside and outside ROI. *p* values are further adjusted by Benjamini and Hochberg method. In the case of multiple datasets representing 2 or more conditions, differential regions are identified by either Kruskal–Wallis or ANOVA statistical tests. In this case, *p* value for each ROI is computed from normalized intensities grouped across different conditions. *p* values are further adjusted by Benjamini and Hochberg method. Normalized read intensities within the significantly differentially enriched regions are then converted into standard z-scores, which are represented as a heatmap. To segregate differential regions specific to any specific dataset, results can be subjected to clustering. If expression data across multiple systems are provided, each differential ROI is assigned to the closest gene, and the distribution of expression of target genes across different clusters is presented as boxplot. Normalized read intensities are computed in similar fashion as explained above but only restricted to ROI not genome wide.

### Selection and classification

Many epigenomic studies involve characterizing and classifying set of ROIs on the basis of some known properties. For such studies, we implemented SVM in our program. It can be used for characterizing two sets of ROI on the basis of any given datasets to determine whether the given datasets are capable of differentiating them. If the datasets are fruitful in the characterization of ROI through this process, the analysis can be further extended in classifying new sets of ROIs using the constructed model. In this version of EpiMINE, we only support classification studies for only two classes. In future, we plan to implement for more than two classes.

If the number of datasets used for characterizing two sets ROIs is too large, the program provides an option to pre-select meaningful datasets. The advantage of pre-selection resides in filtering out datasets that have no or very minimal contribution for the classification. To filter out non-contributing factors, this program uses a recursive feature elimination approach where all possible subsets are considered, and an accuracy score for each best combination is reported. Out of these, variables with the best combination scoring high accuracy are reported. This combination of datasets can now be further used for building the SVM model. This analysis is performed using R package caret.

To build the classification model, the program provides the provision to choose an either linear or nonlinear (radial, Laplacian) classifier. Given two sets of ROIs, a positive set and a negative set of ROIs and *n* different datasets, the program quantifies provided datasets within all ROIs using genome-wide approach (as explained above) and constructs a matrix where columns represent datasets, rows represent ROI and each cell represents normalized intensities. Labels representing each ROI to positive and negative sets are maintained separately. Using the constructed matrix and labels information as training dataset, a classification model is constructed. For constructing strong classification model, we provide option for k-fold cross-validation. Using this approach, the training set is split into *k* groups of approximately the same size; then SVM model is iteratively trained using *k* − 1 groups and simultaneously makes prediction on the group that was left aside. By default, *k* is set to 10. For a given combination of datasets, analysis presents the performance as receiver operating characteristic (ROC) curve, with the true positive rate (TPR) plotted against the false positive rate (FPR). The program lists the area under the ROC curve (AUC) for the classification. The higher the AUC, the greater the possibility of classifying two sets of ROIs. Once the user is satisfied with the classification model based on the training data, the analysis can be further extended to predict a similar classification on a new set of ROIs. Above-explained SVM analysis is performed using R package kernlab.

### Bayesian network

In any given cell type, different HMs and TFs are enriched/bound through the genome. These factors (HMs/TFs) together regulate transcription. Using ChIP-seq from many cell types, the localization of these different factors was mapped throughout the genome. From these data, one can study which different factors function dependently/independently, either genome wide or within a ROI. Such studies can be explored in this program using Bayesian network (BN), which helps to predict probabilistic relationships between a set of different factors. In general terms, for a given finite set of random discrete variables *X* = (*x*1, *x*2, *x*3… *xn*), BN is an directed acyclic graph that signifies joint probability distribution over *X*. Nodes correspond to variables, and edges correspond to the influence of one variable on another. A unique joint probability distribution *P* of *X* can be written as:

$$P\left( X \right) = \mathop \prod \limits_{i = 1}^{n} P\left( {Xi|\varPi Xi} \right)$$

where Π*xi* represent parent(s) of *Xi*.

 Program is fed with *n* different bed files, where each bed file represents one factor. A matrix in discrete format is constructed signifying presence or absence of the factor within the region of analysis. Using such constructed data, a joint distribution model is learned by either constraint- or score-based methods. For constraint-based structure methods, algorithms like grow shrink, incremental association, fast incremental association and interleaved incremental association are supported. Similarly, for score-based structure-learning methods, algorithms like hill climbing and tabu search are supported. In constraint-based learning method, BN is built using conditional independence test, which in our case we use mutual information as test statistic. On the other hand, in score-based learning method, BN is constructed using heuristic optimization approach where candidate BN is assigned with score representing goodness of fit which is then attempted to maximise by the algorithm. We use Bayesian information criterion (BIC) as scoring function for scoring BN. To generate networks with high predictive power, a selected learning method is applied iteratively on randomly selected data (default 90 % per cent) from the original data for 100 times (default). Based on a selected threshold, a probabilistic network is generated with only those edges that are identified at least in 80 % (default) of the generated networks. Above-explained Bayesian network analysis is performed using R package bnlearn.

## Additional file

10.1186/s13072-016-0095-z Contains two supplementary figures and corresponding legends: Figure S1 provides a graphical description of the quantification workflow. Figure S2 provides a comparison of differences in different modes of quantification, annotation of differentially regulated ROI and quantification of different ChIPs in Suz12 binding regions.

## Abbreviations

AUC: area under ROC curve
BAM: binary version of sequence alignment/map (SAM)
BN: Bayesian network
BIC: Bayesian information criterion
ChIP: chromatin immunoprecipitation
CpGi: CpG island
FPKM: fragments per Kb per million
FPR: false positive rate
Gm12878: lymphblastoid
GUI: graphical user interface
H3K27ac: H3K27 acetylation
H1hESC: H1 human embryonic stem cells
HeLaS3: cervical carcinoma cell line
HepG2: liver carcinoma cell line
HM: histone modification
HSMM: skeletal muscle fibroblast cell line
HUVEC: umbilical vein endothelial cell line
K562: leukaemia cell line
NGS: next-generation sequencing
NHEK: epidermal keratinocyte cell line
NHLF: human lung fibroblast cell line
PCA: principal component analysis
PTM: post-translational modification
ROC: receiver-operating characteristic
ROI: regions of interest
RPKM: reads per Kb per million
SVM: support vector machine
TF: transcription factor
TPR: true positive rate
TSS: transcription start site
