# Supplementary material for: EpiMINE, a computational program for mining epigenomic data
Source: Epigenetics Chromatin. 2016 Sep 29;9:42. doi: 10.1186/s13072-016-0095-z (PMC5043526; doi:10.1186/s13072-016-0095-z)
Supplement: Supplementary file 1 — 10.1186/s13072-016-0095-z Contains two supplementary figures and corresponding legends: Figure S1 provides a graphical description of the quantification workflow. Figure S2 provides a comparison of differences in different modes of quantification, annotation of differentially regulated ROI and quantification of different ChIPs in Suz12 binding regions. [file 13072_2016_95_MOESM1_ESM.pdf]

SUPPLEMENTARY FIGURE LEGENDS

Figure S1. Quantification workflow

A. Schematic flow of genome wide based quantification procedure followed by EpiMINE.

Figure S2. Differences in different modes of quantification, annotation of differentially regulated ROI and quantification of different ChIPs in Suz12 binding regions

A. Same as Figure 2C, where intensities are scaled independently for individual sample.
B. Same as Figure 2C but with intensities scaled globally over all samples.
C. Same as Figure 3A but without using strand information.
D. Same as Figure 3B but without using strand information.
E. Tissue specificity of genes whose promoters were differentially regulated in skeletal muscle identified in Figure 4A.
F. Tissue specificity of genes whose promoters were differentially regulated in keratinocytes identified in Figure 4A.
G. Heatmap with intensities presented for Ezh2, Ctbp2 and Suz12 in Suz12 binding regions.

Figure S1

Genome Wide Based Quantification

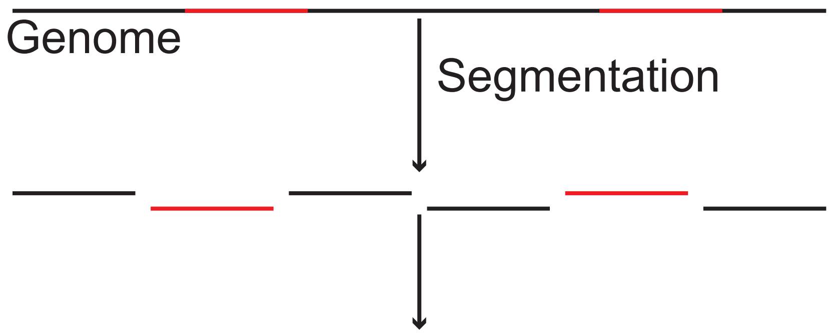

Tag Count in N datasets

Bin ID	Dataset 1	Dataset 2	Dataset 3	Dataset N
1	10	21	8	29
2	1000	13	1215	19
3	20	15	14	8
4	15	900	12	13
5	800	47	17	16
6	12	11	19	1424

Normalize

Bin ID	Dataset 1	Dataset 2	Dataset 3	Dataset N
1	3.93204	8.25728	3.14563	11.40291
2	393.204	5.11165	477.743	7.470873
3	7.86408	5.89806	5.50485	3.145631
4	5.89806	353.883	4.71845	5.11165
5	314.563	18.4806	6.68447	6.291262
6	4.71845	4.32524	7.47087	559.9223

Scale

Independent

Bin ID	Dataset 1	Dataset 2	Dataset 3	Dataset N
1	0	0.01125	0	0.014831
2	1	0.00225	1	0.007768
3	0.0101	0.0045	0.00497	0
4	0.00505	1	0.00331	0.003531
5	0.79798	0.04049	0.00746	0.00565
6	0.00202	0	0.00911	1

Global

Bin ID	Dataset 1	Dataset 2	Dataset 3	Dataset N
1	0.00141	0.00918	0	0.014831
2	0.70056	0.00353	0.8524	0.007768
3	0.00847	0.00494	0.00424	0
4	0.00494	0.62994	0.00282	0.003531
5	0.55932	0.02754	0.00636	0.00565
6	0.00282	0.00212	0.00777	1

Extract Regions of Interest

Bin ID	Dataset 1	Dataset 2	Dataset 3	Dataset N
2	1	0.00225	1	0.007768
5	0.79798	0.040495	0.007457	0.00565

Bin ID	Dataset 1	Dataset 2	Dataset 3	Dataset N
2	0.700565	0.003531	0.852401	0.007768
5	0.559322	0.027542	0.006356	0.00565

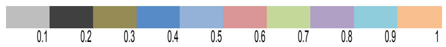

Color Coding

Bin ID	Dataset 1	Dataset 2	Dataset 3	Dataset N
2	1	0.00225	1	0.007768
5	0.79798	0.040495	0.007457	0.00565

Bin ID	Dataset 1	Dataset 2	Dataset 3	Dataset N
2	0.700565	0.003531	0.852401	0.007768
5	0.559322	0.027542	0.006356	0.00565

Figure S2

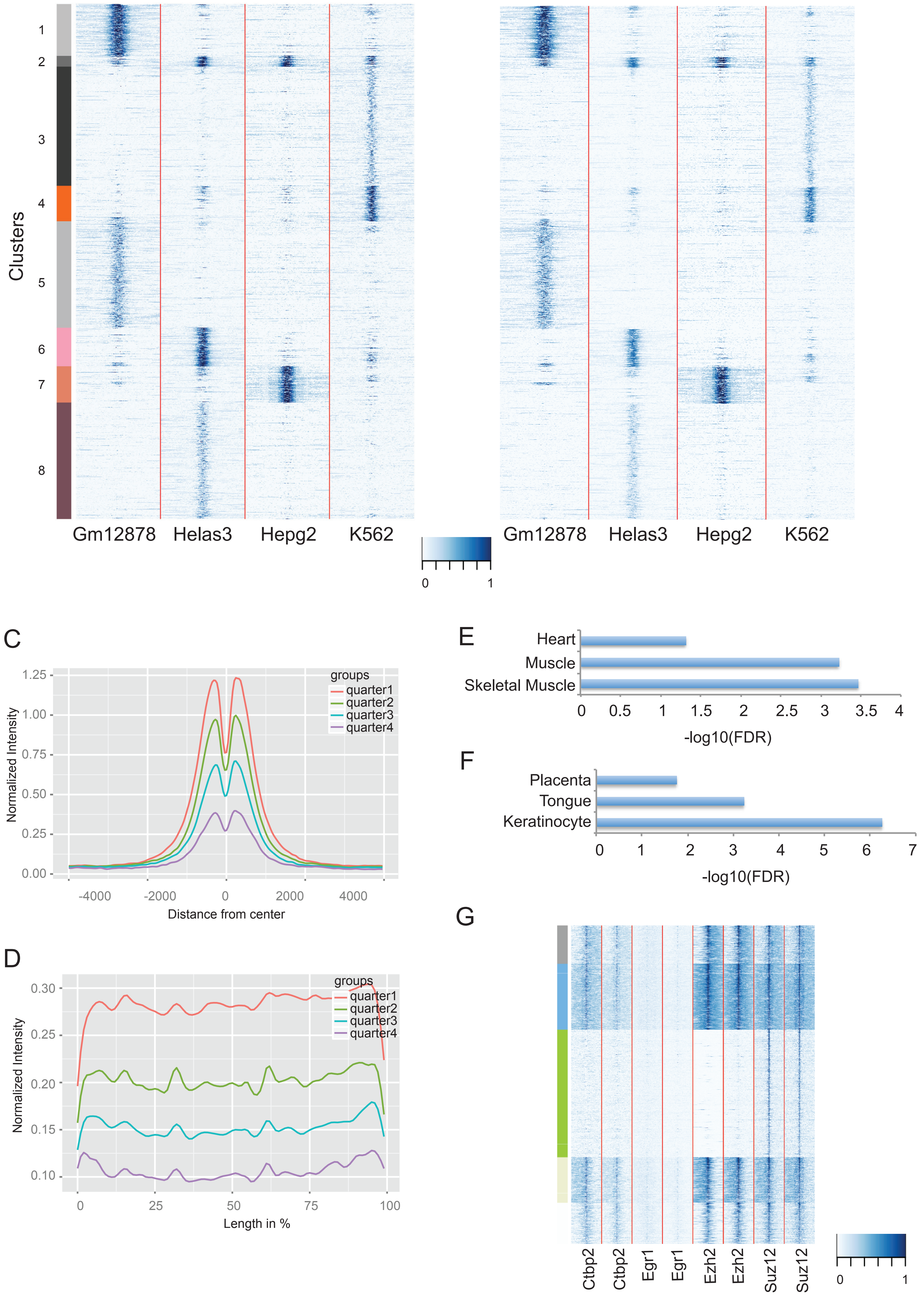
A
B
Clusters
1
2
3
4
5
6
7
8
Gm12878
Helas3
Hepg2
K562
0
1
C
groups
quarter1
quarter2
quarter3
quarter4
Normalized Intensity
Distance from center
D
Length in %
E
Heart
Muscle
Skeletal Muscle
-log10(FDR)
F
Placenta
Tongue
Keratinocyte
G
Ctbp2
Egr1
Ezh2
Suz12
